# The Good, the Bad, and the Unknown: Quality of Clinical Laboratories in Kampala, Uganda

**DOI:** 10.1371/journal.pone.0064661

**Published:** 2013-05-30

**Authors:** Ali M. Elbireer, J. Brooks Jackson, Hakim Sendagire, Alex Opio, Danstan Bagenda, Timothy K. Amukele

**Affiliations:** 1 Makerere University-Johns Hopkins University Clinical Core Laboratory at Infectious Diseases Institute, Kampala Uganda; 2 Department of Pathology, Johns Hopkins University School of Medicine, Baltimore, Maryland, United States of America; 3 Central Public Health Laboratory/Ministry of Health, Kampala, Uganda; 4 Makerere University, College of Health Sciences, Kampala, Uganda; McGill University, Canada

## Abstract

**Background:**

Clinical laboratories are crucial in addressing the high rates of communicable and non-communicable diseases seen in sub-Saharan Africa (SSA). However, the most basic information, such as the number and quality of clinical laboratories in SSA, is not available. The objective of this study was to create a practical method for obtaining this information in SSA towns and cities using an initial survey in Kampala, Uganda.

**Methods:**

Kampala city was divided into 5 partially-overlapping regions. Each region was assigned to 2–3 surveyors who identified and surveyed laboratories in their respective regions; in person and on foot. A modified version of the World Health Organization - African Region (WHO/AFRO) Laboratory Strengthening Checklist was used to obtain baseline measures of quality for all clinical laboratories within Kampala city. The surveyors also measured other attributes of each laboratory, such as their affiliation (government, private etc), designation (national hospital, district hospital, standalone etc), staff numbers, and type of staff.

**Results:**

The survey team identified and surveyed 954 laboratories in Kampala city. 96% of laboratories were private. Only 45 (5%) of the laboratories met or surpassed the lowest quality standards defined by the WHO/AFRO-derived laboratory strengthening tool (1-star). These 45 higher-quality laboratories were, on average, larger and had a higher number of laboratory-specific staff (technologists, phlebotomists etc) than the other 909 laboratories. 688 (72%) of the 954 laboratories were not registered with the Ministry of Health (MoH).

**Conclusions:**

This comprehensive evaluation of the number, scope, and quality of clinical laboratories in Kampala is the first published survey of its kind in sub-Saharan Africa. The survey findings demonstrated that laboratories in Kampala that had qualified personnel and those that had higher testing volumes, tended to be of higher-quality.

## Introduction

Sub-Saharan Africa (SSA), with a population of approximately 860 million people, has high rates of communicable disease as well as rates of non-communicable disease that are rapidly approaching those seen in more affluent sedentary societies.[1]–[3] Clinical laboratories are a critical part of addressing these challenges because they are a basis of clinical decision making. However the most basic information, such as the number and quality of clinical laboratories in many SSA countries, is not available because of two challenges. First, data on the number and quality of labs are not available in settings without comprehensive registration of laboratories or a single payer system. Second, until recently, there was no obvious tool to measure the quality of clinical laboratories in resource-limited settings.

There are two widely acceptable international accreditation standards for laboratory quality. The 1988 Clinical Laboratory Improvement Amendments of the United States congress [4] and ISO 15189, the Clinical-Laboratory standards of the International Society for Standardization. [5] Most laboratories in developing countries fall so far short of these accreditation standards that they cannot currently make any realistic attempts at accreditation. In recognition of this fact, the World Health Organization – Africa regional office (WHO-AFRO) created a Step-wise Clinical Laboratory Accreditation scheme to measure quality improvements in laboratories that are not yet accredited. [6].

This WHO-AFRO stepwise accreditation scheme is based on four core criteria plus 12 quality system essentials (QSE) which are derived from ISO 15189 standards. [7] ^,^ [8] The four core criteria are an 80% compliance with stated Turnaround Times, a sufficient Volume of Testing to maintain staff competency, performance of daily Internal Quality Control and an 80%, 2-cycle pass rate on External Quality Control. The 12 QSE’s are, Documents and Records; Facilities and Safety; Equipment; Purchase and Inventory; Process Control; Assessment; Personnel; Customer Service; Occurrence Management; Process Improvement; Information Management; and Organization. Conformity to the QSEs are based on a checklist. Checklist scores of 55–64%, 65–74%, 75–84%, 85–94% and >95% are ultimately translated into a 0- to 5-star scale. [6].

**Figure 1:**
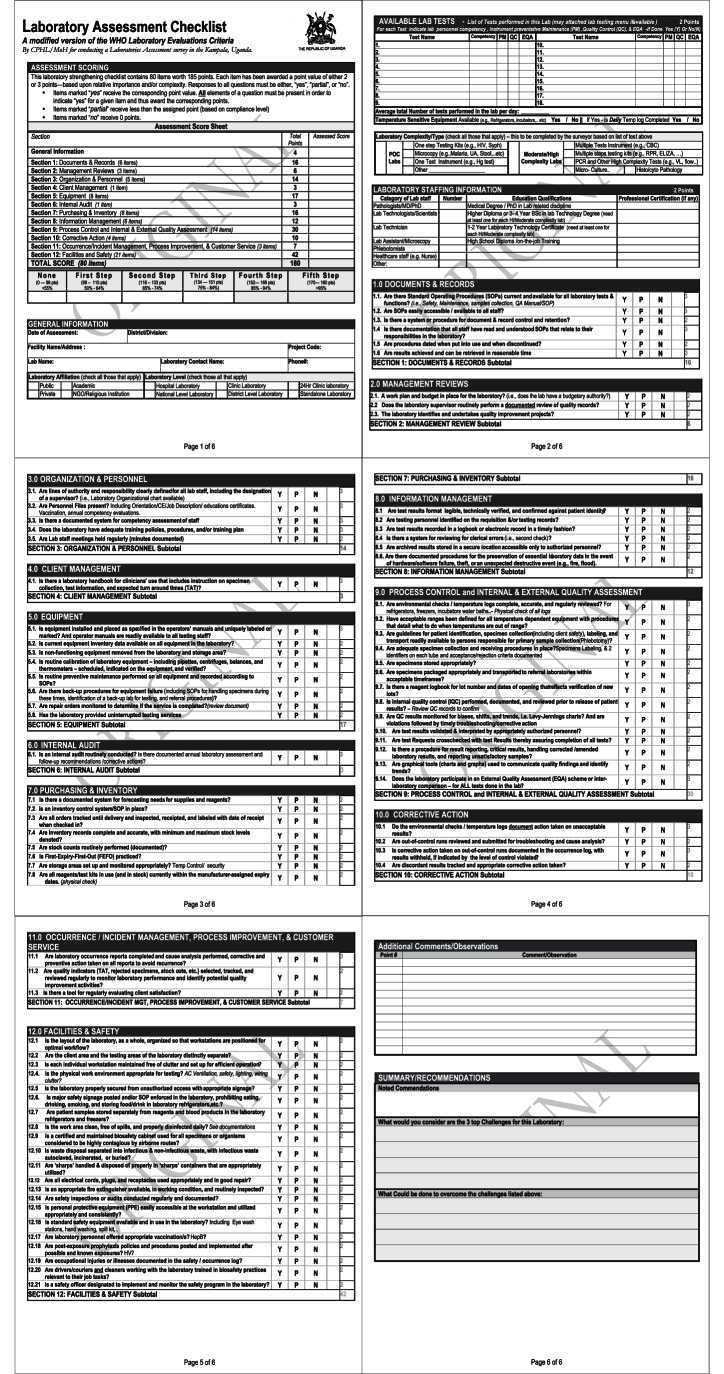
The modified version of the WHO/AFRO Laboratory Strengthening Checklist.

A cross-sectional survey of all laboratories was performed during the last quarter of 2011. A modified version of the WHO/AFRO Laboratory Strengthening Checklist (Figure1) was used to obtain baseline measures of quality for all clinical laboratories identified within Kampala. Previous work on the quality of clinical laboratories in SSA has been mostly descriptive, [9] based on single tests, [10] or focused on a few target laboratories. [11], [12] In order to fill the information gap regarding clinical laboratories in SSA, the study investigators created a scheme for comprehensively identifying and assessing the quality and scope of clinical laboratories in large towns and cities. The initial rollout of this scheme was in the city of Kampala, the capital of Uganda.

## Methods

### Organization of the Survey

This project was a collaboration between the Ministry of Health (MoH), the Central Public Health Laboratories (CPHL), the Allied Health Professional Council (APHC), and individuals from the Makerere University-Johns Hopkins University laboratory. The full team was comprised of a project Director, a project Coordinator, representatives from the MoH, CPHL, APHC, as well as 13 surveyors. The surveyors comprised the survey team and were clinical laboratory professionals with practice and audit experience. The survey team attended two training sessions, in a College of American Pathologists (CAP) accredited laboratory. The team also participated in a practicum designed to improve assessment skills and minimize inter-observer variability. Briefly, the training sessions included role-play as well as reviews of Good Clinical Laboratory Practices (GCLP), the checklist, and various assessment skills such as probing, observation, and judgement.

### Design of the Survey Tool

The WHO/AFRO laboratory strengthening tool is comprised of 110 checklist questions worth 250 points, which requires extensive training to administer properly. Each of the 110 checklist questions are differentially weighted, and each question can have several sub-items. Specific action items needed to comply with the requirements of the checklist are varied based on the complexity of the site being surveyed (community level, district level, regional or provincial level and central level). The scope of our task and the limited number of surveyors necessitated the design of a modified version of the WHO/AFRO quality assessment checklist that would take less time (i.e., 30–40 minutes) per site to survey (Figure 1). Also, the modified WHO/AFRO is composed of the same 12 QSEs that make-up the full WHO/AFRO checklist.

The modified checklist used for this survey was comprised of 80 ‘yes’/‘no’ questions Each item was awarded a point value of either 2 or 3 points based on its relative importance and complexity, for a total of 185 possible points. Checklist scores of 55–64%, 65–74%, 75–84%, 85–94% and >95% are ultimately translated into a 0- to 5-steps (rather than stars) scale. This modification was done to avoid confusion with the original WHO-AFRO laboratory strengthening tool. However, in this paper we use the term “star” for standardization purposes.

### Rollout of the Survey

Kampala is the largest city and capital of Uganda with an area of 73 square miles. In 2011, the Uganda Bureau of Statistics (UBOS) estimated the mid-year population of Kampala city at 1,659,600. Kampala city is composed of five geographic and administrative sub-divisions. The 13-member survey team was divided into 5 groups of 2–3 individuals each. Each group was responsible for assessing each of the five sub-divisions of Kampala city. The areas assigned to each group were organized so there was a slight overlap between groups. This redundancy was introduced as an internal quality tool to allow us to evaluate survey completeness and consistency.

Clinical laboratories were defined as all establishments where laboratory tests are performed on human specimens for the purpose of healthcare. In order to capture all such establishments, the survey included both sites self-identified as laboratories (so-called standalone), as well as those embedded within healthcare establishments and only identified by the survey team. For each laboratory thus identified, the survey team documented the number and complexity of tests performed; the affiliation (government, private etc); designation (national hospital, district hospital, standalone etc); staffing; as well as baseline measures of quality and safety. Quality was assessed using a modified version of the WHO-AFRO laboratory strengthening and assessment tool. The questions were administered in-person, to the ‘in-charge’ or designee at each laboratory facility. Each survey was conducted in English over a period of 30–45 minutes. All elements of a question had to be present in order to indicate ‘yes’ for a given item. Items marked ‘partial’ received a percentage of the assigned point based on the compliance levels. Items marked ‘no’ received zero points. Laboratories that achieved an assessment score of ≥55% were also given the appropriate 0 to 5-star rank. [6].

Since most private laboratories in Kampala were neither registered nor regulated it was important for the survey team to categorize each laboratory based on affiliation and type. Laboratory types were defined as follows.

- Stand-alone laboratories - Not affiliated with a specific medical facility.
- Clinic laboratories - Operated by, and serving a non-hospital facility in which patients receive medical or surgical care.
- National laboratories -Disease-specific reference laboratory for the country e.g. the National Tuberculosis (TB) reference laboratory.
- Hospital - Operated by, and serving a hospital facility.
- District - Owned and/or operated by the government, and associated with a regional referral healthcare centre.

Laboratory affiliations were defined as follows:

- Public - Owned and/or operated by the government.
- Private - For-profit laboratories owned by one or a group of individuals.
- Academic - Primarily carry out academic research and teaching.
- Non-Governmental Organizations (NGO)- Owned by legally constituted organizations operating independently from government.
- Religious - Owned, funded or operated by religious foundations or groups.

Even though some of the laboratories met multiple criteria, they were classified and analysed according to their primary affiliation. NGO and religious-affiliated laboratories were analysed together.

## Results

### Number and Quality of Clinical Laboratories

The survey team identified 954 working clinical laboratories within the Kampala city limits. 909 (95.3%), 14 (1.5%), and 14 (1.5%), laboratories had a zero-, 1- and 2-star ranking respectively (Table 1). 7 (0.7%), 6 (0.6%),and 4 (0.4%) laboratories had a 3-, 4- and 5-star ranking respectively (Table 1). Only 28% (266 of 954) of the laboratories identified in Kampala were registered with the Ministry of Health. There was no significant difference in the checklist score (i.e., quality of laboratories services) of registered (median score 35, IQR 4-174) versus unregistered (median score 32, IQR 0-180) laboratories.

**Table 1:** Relationship between laboratory quality (WHO-AFRO stars), the average number of laboratory-specific staff per laboratory, and the median laboratory testing volume.

No. of Stars	Percentage of labs in star cohort	Median no. of daily tests per lab
**0**	95.3%	5
**1**	1.5%	20
**2**	1.5%	100
**3**	0.7%	200
**4**	0.6%	140
**5**	0.4%	200

### Clinical Laboratory Testing Volume versus Quality

Table 1 shows the laboratories in Kampala city grouped by quality, versus the median number of tests per laboratory. Laboratories that had higher testing volumes had higher quality scores. The number of tests per laboratory was 5, 20, 100, 200, 140, and 200 for the 0-, 1-, 2-, 3-, 4- and 5-star laboratories, respectively.

### Clinical Laboratory and Staff versus Quality

Table 2 shows the laboratories in Kampala city grouped by quality, versus the number of lab-specific staff per laboratory. Table 2 also shows the laboratories in Kampala city, grouped by quality, versus the type of staff per laboratory. Each cell shows the average numbers of each type of staff (technician, phlebotomist etc.) found in laboratories of each quality (star) group. In addition, each cell shows the relative number of each type of staff per laboratory. This grouping is the most direct approximation of the relative number of each type of staff found in each quality (star) group.

**Table 2:** Relationship between laboratory quality (WHO-AFRO stars), staff type, and staff number.

	Laboratory quality versus Average number of various laboratory-staff per lab				
No. of Stars	Technologists (N)	Technicians (N)	Assistants (N)	Phlebotomists(N)	Total # of Lab-specific staff (N)
Zero	0.18	0.4	0.4	0	1
	(163)	(401)	(316)	(20)	(900)
One	0.9	1.6	0.4	0	3
	(13)	(23)	(6)	(0)	(42)
Two	0	2.9	2.3	0	5
	(0)	(44)	(35)	(0)	(79)
Three	3.5	2.5	0.2	0	6
	(21)	(15)	(1)	(0)	(37)
Four	2.8	3	0.3	0.75	7
	(11)	(12)	(1)	(3)	(27)
Five	7.3	3	4.3	2	19
	(22)	(15)	(13)	(6)	(56)

The number of laboratories with at least one laboratory-specific staff member is shown in brackets.

The absolute and relative number of laboratory-specific staff (laboratory technologists, laboratory technicians, laboratory assistants and phlebotomists) are all positively related to improved laboratory quality (number of stars). In 44 of these 45 higher-quality laboratories, there was at least one laboratory-specific (technologists, phlebotomists etc.) staff member. The average number of laboratory-specific staff members was 4.4-per-lab for these 45 laboratories which scored >1-star, versus 1-per-lab for the 911 laboratories which scored zero-stars. The average number of these laboratory-specific staff members per laboratory was 3, 5, 6, 7, and 19 for the 1-, 2-, 3-, 4- and 5-star laboratories respectively. The relative number of physicians or other healthcare staff did not demonstrate any clear relationship to laboratory quality.

### Clinical Laboratory Affiliation versus Quality

Table 3 shows the numbers and quality of laboratories in Kampala city, grouped by affiliation. 915 (95.9%) laboratories were private, 23 (2.4%) were Public, 14 (1.5%) were NGO/Religious, and 2 (0.2%) were Academic Labs. All the academic laboratories (100%), most of the NGO/religious laboratories (71%) and the majority of private laboratories (97%) were zero-star quality laboratories. Conversely, more than two-thirds of the public laboratories were greater than zero-star quality rating. Overall, public laboratories had higher quality scores than private laboratories.

**Table 3:** Relationship between laboratory quality (WHO-AFRO stars) and laboratory affiliation.

	Laboratory quality versus Laboratory Affiliation				
	Academic	NGO/Religious	Public	Private	Totals
0-stars	2	10	7	890	909
1 to 5-stars	0	4	16	25	45
Column Totals	2	14	23	915	954

### Clinical Laboratory Type versus Quality

Table 4 shows the numbers and quality of laboratories in Kampala city, grouped by type. 895 (93.8%) were clinic laboratories, 34 (3.6%) were Hospital, 16 (1.7%) were National Reference, and 9 (0.9%) were Stand-alone laboratories. Overall the Hospital, National referral, and Stand-alone laboratories had higher quality scores than private laboratories.

**Table 4:** Relationship between laboratory quality (WHO-AFRO stars) and laboratory type.

	Laboratory quality versus Type of laboratory				
	Clinic	Hospital	National Ref.	Stand-alone	
0-stars	882	20	2	5	909
1 to 5-stars	13	14	14	4	45
Column Totals	895	34	16	9	954

## Discussion

To the best of the authors’ knowledge, this report is the first published comprehensive cross-sectional quality survey of all clinical laboratories in a sub-Saharan African city. The survey team identified a total of 954 clinical laboratories in Kampala city. This number was more than three times the number of laboratories that were previously registered with the MoH. This finding of an additional 688 laboratories demonstrates the power of the active discovery methods used in this study. The laboratories were identified by searching in person, not passively using records. Of note, quality scores of the 28% of laboratories that were registered with the Ministry of Health were not different from unregistered laboratories and demonstrates that registration or other forms of identification have minimal impact without active regulatory oversight.

Only 45 (4.7%) of the 954 laboratories in Kampala city met the lowest quality standard (1-star) of the modified WHO-AFRO laboratory-strengthening instrument. The main determinants of quality in this setting appeared to be laboratory size and staff training. Larger laboratories, such as public laboratories, had higher quality scores than private laboratories. The relationship between laboratory size and quality was present whether size was measured in terms of number of daily tests or number of staff. This relationship between laboratory quality and laboratory size is similar to what has been seen in other countries prior to the advent of laboratory regulations. [13], [14] The authors are not aware of any published studies that have attempted to determine the causes of this phenomenon. However, larger laboratories have higher workloads and tend to be associated with ministries of health, [14], [15] and so may have more oversight and more external funding. For example, the U.S. President’s Emergency Plan for AIDS Relief (PEPFAR) is America’s commitment to fighting the global HIV/AIDS pandemic and represents approximately $46 billion to bilateral HIV/AIDS programs, the Global Fund to Fight HIV/AIDS, Tuberculosis and Malaria, and bilateral TB programs through fiscal year (FY) 2010. ^16^ In 2009, PEPFAR dedicated $1.0 to $1.4 billion to supporting health systems, of which 6% was earmarked for strengthening laboratory systems.^16^ Thus the higher quality in public laboratories could be attributed to the financial resources given to the Ministry of Health by these international donors for strengthening national laboratory systems in SSA including Uganda.

An education in laboratory science alone did not guarantee even the lowest level of laboratory quality. 79% of laboratory-specific staff worked in zero-star laboratories. These findings are consistent with previous studies of the behaviour of healthcare professionals which demonstrated that even when the educational intervention is intense and sustained for up to 2 years, education and guidelines alone are weak interventions and produce small changes.[16]–[18] Education only guarantees consistent quality when paired with strong, multifactorial interventions such as financial and regulatory systems that reward quality outcomes. [17].

The survey findings reveal three areas in which focused interventions could significantly improve quality at low or no additional cost in this SSA setting. First, clinical laboratory work should be conducted by people trained to work in clinical laboratories. Second, all laboratories should perform test volumes that are high enough to support staff competency. Thus, creating central laboratory hubs in strategic locations to help consolidate diagnostic services and to provide support and oversight to other small laboratories, are likely to improve the quality of diagnostic testing. Third, registration of a laboratory with the Ministry of Health should be tied to clearly defined quality standards and accountability if registration is to signify a laboratory of good quality. Finally, even though this survey didn’t specifically evaluate the impact of the presence of trained laboratory managers on quality, previous experience suggests that trained laboratory professionals, including managers, are important in ensuring quality laboratory outcomes. [19].

The first step in improving the quality of clinical laboratories in SSA is to determine the identity and quality of laboratories in a comprehensive way. This survey provides a model for obtaining the data that are required to strengthen clinical laboratory quality in Uganda and other SSA countries.
